# The influence of selected anthropometric parameters on psychomotor abilities among professional Rugby Union players

**DOI:** 10.1186/s13102-023-00735-y

**Published:** 2023-09-29

**Authors:** Maciej Śliż, Wojciech Paśko, Bartosz Dziadek, Łukasz Godek, Katarzyna Bliźniak, Élvio Rúbio Gouveia, Krzysztof Przednowek

**Affiliations:** 1 Institute of Physical Culture Sciences, Medical College of Rzeszów University, University of Rzeszów, Rzeszów, Poland; 2 Department of Physical Education and Sport, University of Madeira, Funchal, Portugal

**Keywords:** Reaction time, Movement time, Psychomotor computer tests, Polish National Rugby Team, Professional players

## Abstract

**Background:**

One of the most popular varieties of rugby is Rugby Union, in which a team consists of 15 players. Rugby Union is a full-contact sport, and players must demonstrate strength, endurance, speed and agility. During the match, players participate in multiple physical collisions and tackles, short-duration and high-intensity sprinting efforts. In addition, one of the elements affecting the effectiveness of the player’s game can be the reaction time of the rugby player to the visual stimulus and the ability to read the game and react to the situations on the pitch. The level of psychomotor abilities of a person or a player practising sports can be influenced by various factors, including age, body height, body weight, type of sport practised or level of training. The study aimed to analyse the psychomotor abilities of professional rugby union players, investigate the relationship between the selected anthropometric characteristics and psychomotor abilities, and compare obtained results with the control group.

**Methods:**

The study covered 22 players of the Polish National Team (age: 29.3 ± 5.4) and 27 students in the control group (age: 24.3 ± 3.9). The tests were carried out using the Test2Drive computer system of tests. For psychomotor abilities analysis, four tests were used: Simple Reaction Test, Choice Reaction Time Test, Hand-Eye Coordination Test and Spatial Orientation Test. The statistical analysis compares groups using basic statistical measures, and statistically significant differences between groups were checked. In addition, multiple linear regression was used.

**Results:**

The analysis showed statistically significant differences between the groups in the simple reaction time test and the movement time for the test assessing reaction time with choice and eye-hand coordination. Multiple regression analysis conducted for both groups showed a statistically significant influence of some anthropometric parameters on the examined psychomotor abilities. The calculated multiple regression models had a high fit.

**Conclusions:**

The analysis showed that professional Rugby Union players have shorter movement time than the control group. For reaction time, statistically significant differences were observed only for simple reaction time. Moreover, linear regression analysis showed that body height and weight affect the selected psychomotor abilities.

## Background

Reaction time can be a key factor in success in many sporting competitions. Kida et al. [1], Grigore et al. [2], and Paul et al. [3] proved that psychomotor abilities including reaction time can play a key role and lead to victory. Moreover, a very small difference in the players’ reaction time plays a significant role in distinguishing between the winner and loser [4]. In team sports, reaction time is the ability to respond as quickly as possible to stimuli like sound, light, and signal [5]. In the literature, reaction time is becoming increasingly important, especially in a team sport where not only straight ahead speed is an important but mainly quick reaction to the situation on the pitch [5]. According to Atan and Akyol [6], athletes who practice team sports should have certain psychomotor abilities. Team sports, such as rugby, have been described as activities that require a mixed effort (aerobic-anaerobic) depending on different moments of the game. Therefore, we can say that a good development of the psychomotor abilities of players combined with good physical training specific to each position on the field can lead to optimal manifestations of the players’ skills during matches and implicitly lead to obtaining the desired performance results [7].

Grigore et al. [2] show that systematically practising sports where there is direct contact with an opponent is an effective way of developing abilities associated with increased efficiency and eye-hand coordination at low and high speeds. There is a close relationship between perception and activity in people actively practising sports; in the foretime, limited tasks sports require players to extract the most valuable source of visual information and use this information to quickly predict the outcome of an opponent’s move [8]. The ability to anticipate the movement of the opponent and ball, reaction to pitch situation, decision-making, speed of perception, and a high level of sensory efficiency and motor abilities have an impact on the achievement of sports success [3, 9, 10].

One of the most popular team sports in the world is rugby, commonly known as rugby union [11], where reaction time is considered critical for successful performance. Rugby is a full-contact, high-speed sport where strength, endurance and agility play a huge role in sports results [12–16]. The rugby game is physically demanding, requiring players to participate in multiple physical collisions and tackles, as well as short duration, high-intensity sprinting efforts and reaction time with anticipation during rugby match [17–21]. Compared to most other sports, passing in rugby is unusual as passes must be orientated backwards, contrary to the overall intention of the game, which is to move forwards [22]. This sport discipline is based on technical, tactical, motor and psychomotor preparation [23].

Sight is one of the main sensory systems that involves the implementation of field situations during rugby matches [24]. Rugby players respond mainly to visual impulses. The reaction time of the rugby player to the visual stimulus can affect the ability to read the game and react to the situations on the pitch [23]. According to Gavkare, Nanaware, and Surdi, in a team game, reaction time determines how successful a player is in defense. When an attacking player makes a move or faints, little difference between a slow and a fast reaction by the defensive player determines his success or failure [25]. Thus, as demonstrated by Gabbett, Benton [18] and Serpell et al. [26], faster decision and movement times in the reactive agility test, which was based on visual stimuli, can characterise more highly skilled players or the elite group of rugby league.

Rugby players at the highest level of experience should be characterized by a high level of reaction time, the correctness of decision-making, eye-hand coordination, spatial orientation and anticipation in relation to non-players of sports amateurs [27]. In addition, one of the elements that can have an impact on psychomotor abilities may be body height [28], body mass, BMI [29–31] or age [32, 33]. Samaras [28] pointed out that the height of the body, and thus the path that the nerve impulse must travel from the brain to, for example, the limbs, may affect the reaction time speed. The age of the subjects may also influence the reaction time [32], the significance of which was examined by Tønnessen in a group of 1319 sprinters [33]. The increased reaction time may also result in higher BMI and body mass, as shown in a study by Nikam et al. [29], Skurvydas et al. [30] and Paśko et al. [31].

The purposes of this study were threefold: (1) to describe the psychomotor abilities of male rugby players from the Polish National Rugby Team; (2) to investigate the relationship between anthropometrics characteristics and psychomotor abilities; and (3) to compare the differences in the psychomotor abilities between professional rugby players and students of physical education.

## Methods

### Sample

The study covered 22 Rugby Union players from the Polish National Team (age: 29.3 ± 5.4), while the control group consisted of 27 students (age: 24.3 ± 3.9). Table 1 shows the characteristics of rugby players and the control group. The research was conducted out on rugby players during National Training Camp in October 2020. The control group consisted of physical education students who were in the final year of their master’s studies. All students were physically active and practiced amateur sports. Each of the participants consented to participate in the research. To enter the study, each participant had to be an adult male with no health problems. No one was treated pharmacologically during the study. Research for Rugby students and players has been conducted under similar conditions. The scope and project research was evaluated by the Ethics Committee of the University of Rzeszow / Poland (resolution 10/02/2020).

**Table 1:** Characteristic of Rugby Union players

Group	Rugby	Control	Total
N	22	27	49
Age	$29.3 \pm 5.4$	$24.3 \pm 3.9$	$26.5 \pm 5.2$
Body Height (cm)	$186.0 \pm 7.0$	$180.6 \pm 6.7$	$183.0 \pm 7.3$
Body Weight (kg)	$103.1 \pm 15.2$	$78.9 \pm 9.1$	$89.8 \pm 17.1$
BMI	$29.3 \pm 4.6$	$24.2 \pm 2.1$	$26.5 \pm 4.3$

### Methods

**Fig. 1:**
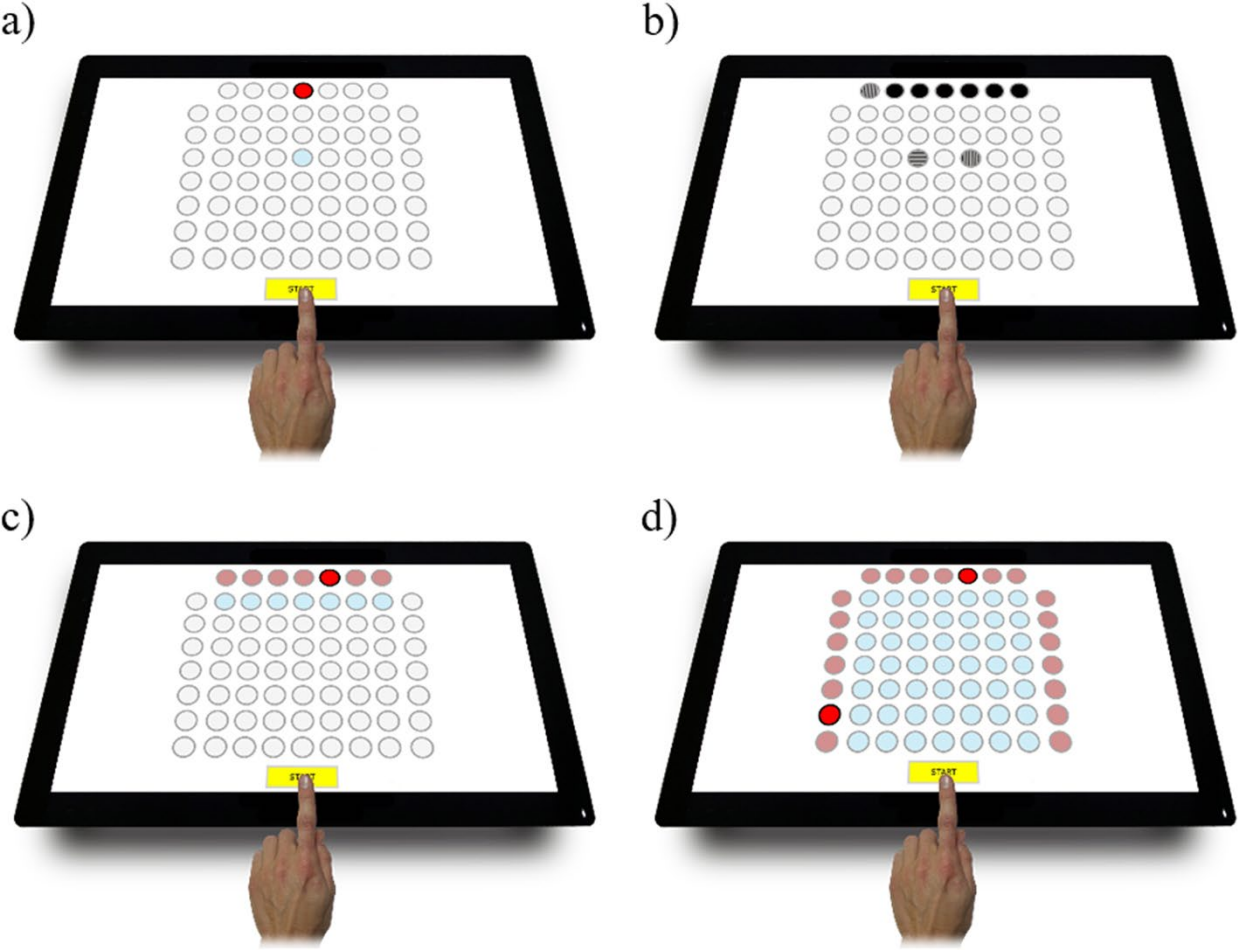
Reaction panel of the Test2Drive system; **a**) SIRT–Simple Reaction Time Test, **b**) CHORT–Choice Reaction Time Test, **c**) HECOR–Hand-Eye Coordination Test, **d**) SPANT–Spatial Anticipation Test

The Test2Drive computer diagnostic system was used to measure and evaluate the selected psychomotor abilities. The Test2Drive computer test battery has been validated by the software manufacturer [34] and fulfills all the requirements of the Ministry of Health Regulation. The system provided a set of four tests to measure simple reaction time (SIRT), choice reaction time (CHORT), hand-eye coordination (HECOR), and spatial anticipation (SPANT). The measurement station and the appearance of the screens are shown in Fig. 1.

### Design and procedures

Four tests of the Test2Drive were used in the study to assess the level of psychomotor abilities [34]. Each of the tests involved the measurement of the reaction time (RT) and movement time (MT). Reaction time (RT) was measured from the appearance of the visual stimulus until the finger was removed from the “START” field. The movement time (MT) was measured from the moment when the finger was moved from the “START” field to the answer selection. The study subjects performed tasks in a room facilitating concentration, in a standing position, using the dominant hand’s index finger (Fig. 1). The temperature during the studies was 20$^{\circ }$ Celsius. Each study test was preceded by a trial test that gave the subject a chance to become acquainted with the test procedure. Each participant has performed the tests in the following order:

- Test SIRT – The measurement is used to evaluate the simple reaction time (Fig. 1a).
- Test CHORT – The measurement is used to evaluate the response time with a choice (Fig. 1b).
- Test HECOR – The measurement was used to assess hand-eye coordination (Fig. 1c).
- Test SPANT – The measurement was used to assess hand-eye coordination with the use of complex spatial information (Fig. 1d).

The full description of the selected tests and the method of conducting the study have been described in previous publications [10, 35].

### Statistical method

The study used basic statistical measures (frequencies, average, median, standard deviation) to assess selected psychomotor abilities considering each group. In addition, the normal distribution was verified using the Shapiro-Wilk test. In the case of normal distribution ($p>0.05$), the statistical significance of differences between individual groups was carried out using the t-student test, while the normal distribution deviated from the Gaussian curve ($p<0.05$), the Mann-Whitney U test was used. The next step was to use a multiple linear regression model. The following values were determined: unstandardized beta ($\beta$), standard error (SE), Residual Standard Error (RSE), the ratio of the mean regression sum of squares divided by the mean error sum of squares (F), multiple R-squared (m. $R^2$) and statistical probability (p).

## Results

The Shapiro-Wilk test showed that the variables CHORT c.r., HECOR MT and SPANT c.r. deviate from the Gaussian curve, therefore a non-parametric test (Mann-Whitney U test) was used to check the test probability. For the remaining variables, a parametric test (t-test) was used. Table 2 shows the results of psychomotor tests of rugby players and the untrained control group. Rugby players in the SIRT test had a longer reaction time (356.0 ± 34.4 ms) than the control group (332.4 ±41.7 ms). In the case of movement time, rugby players have a shorter time (158.1 ± 38.0 ms). The differences between reaction time and movement time in the SIRT test were statistically significant. The CHORT test showed that the rugby group is characterized by both shorter reaction time and movement time. Statistically significant differences were observed only for movement time ($p<0.05$). In the hand-eye coordination test (HECOR), the control group had a shorter reaction time (400.6 ± 51.6 ms), while rugby players had a shorter movement time (206.3 ± 31.4 ms). Statistically significant differences between the groups were found only in movement time. In the SPANT test, the control group also had a shorter reaction time, and the rugby players had a shorter movement time. No statistically significant differences were observed. In addition, the percentage of correct answers was analyzed in the CHORT and SPANT tests. In both cases, the control group was characterized by higher correctness of answers. No statistically significant differences were found.

**Table 2:** Numeral characteristics of psychomotor abilities of groups

Variable	Rugby players		Control group		
	$\bf\overline{x}$ ± sd	*Me*	$\bf\overline{x}$ ± sd	*Me*	*p*
**SIRT**					
RT [ms]	$356.0 \pm 34.4$	357.0	$332.4 \pm 41.7$	328.0	0.0389*
MT [ms]	$158.1 \pm 38.0$	156.5	$192.1 \pm 47.4$	182.0	0.0089*
**CHORT**					
RT [ms]	$675.8 \pm 75.9$	676.5	$681.3 \pm 68.6$	678.0	0.7907
MT [ms]	$170.3 \pm 42.4$	162.5	$214.7 \pm 48.0$	223.0	0.0014*
c.r. [%]	$90.7 \pm 16.9$	96.0	$93.6 \pm 5.1$	96.0	0.4214
**HECOR**					
RT [ms]	$417.3 \pm 33.3$	418.0	$400.6 \pm 51.6$	401.0	0.1949
MT [ms]	$206.3 \pm 31.4$	202.0	$243.0 \pm 50.9$	234.0	0.0065*
**SPANT**					
RT [ms]	$616.6 \pm 31.4$	614.5	$570.2 \pm 84.5$	555.0	0.0827
MT [ms]	$227.3 \pm 43.5$	225.0	$242.7 \pm 52.7$	232.0	0.2778
c.r. [%]	$92.7 \pm 11.4$	95.0	$95.9 \pm 5.7$	100.0	0.1565

SIRT – Simple Reaction Time; CHORT – Choice Reaction Time;

HECOR – Hand-Eye Coordination Test; SPANT – Spatial Anticipation Test;

RT – reaction time; MT – movement time; c.r. – correct responses; p – statistical probability; * – $p<0.05$

The next stage of the analysis was a specific assessment of the influence of the following factors such as age, height, body weight and BMI on psychomotor abilities. Tables 3 and 4 show the linear regression model for individual variables for rugby players and control group. Each regression model showed statistical significance ($p<0.05$) for both rugby players and control group. In addition, a very high model fit (m. $R^2$) was observed in each regression model, which was above 95%. Analysis of rugby players showed that body height is a statistically significant predictor variable in the SIRT RT test. This means that for every one unit increase in body height, the reaction time in SIRT test increases by 1.66 ms. The calculated coefficient has a standard error of 0.30 ms. Body height is also a significant statistical predictor of reaction time and the number of correct answers in CHORT test. An increase in body height increases the reaction time ($\beta$ = 2.15 ms) and increases the number of correct answers ($\beta$ = 0.87%). The standard error for the height predictor variable (SE) for the CHORT RT test is 0.60 ms, while for the CHORT c.r. it is 0.12%. In HECOR test, body height is a statistically significant predictor variable for both reaction time and motor time. The ($\beta$) value for HECOR RT is 2.32ms and the standard error is 0.30ms. For HECOR MT, the ($\beta$) factor is 0.80 ms with a standard error of 0.27 ms. For correct answers, statistically significant predictor variables in SPANT c.r. test were observed for body height and body weight. The number of correct answers increases by 0.80% for a 1 cm increase in body height, and ($\beta$) has an error of 0.08%. A 1 kg decrease in body weight results in an increase in SPANT c.r. of 0.65%, with a standard error of 0.28%.

**Table 3:** Multiple Linear Regression for rugby players

Variable	Age		Body height		Body weight		BMI		RSE	m. *R*^2^	F	p
	*β*	SE	*β*	SE	*β*	SE	*β*	SE				
**SIRT**												
RT [ms]	2.11	1.59	1.66***	0.30	-0.79	1.10	2.27	3.15	34.15	0.99	598.40	0.0001
MT [ms]	2.11	1.77	0.38	0.34	0.20	1.23	0.18	3.51	38.05	0.95	95.63	0.0001
**CHORT**												
RT [ms]	4.82	3.16	2.15**	0.60	0.90	2.19	1.43	6.26	67.82	0.99	548.30	0.0001
MT [ms]	3.17	1.86	0.20	0.36	0.66	1.29	-0.95	3.69	35.95	0.96	101.30	0.0001
c.r. [%]	-0.03	0.64	0.87***	0.12	-0.53	0.45	-0.56	1.27	13.79	0.98	241.20	0.0001
**HECOR**												
RT [ms]	-0.22	1.59	2.32***	0.30	-0.88	1.10	2.78	3.16	34.18	0.99	820.20	0.0001
MT [ms]	1.16	1.43	0.80**	0.27	0.61	0.99	-1.38	2.83	30.68	0.98	249.50	0.0001
**SPANT**												
RT [ms]	5.99	3.69	0.81	0.71	1.40	2.56	5.01	7.31	79.16	0.99	337.30	0.0001
MT [ms]	2.46	2.01	0.65	0.38	-0.39	1.39	2.57	3.98	43.16	0.97	153.40	0.0001
c.r. [%]	-0.01	0.41	0.80***	0.08	-0.65*	0.28	0.35	0.80	8.71	0.99	628.10	0.0001

$\beta$ – unstandardized beta; SE – standard error; RSE – Residual Standard Error;

F – the ratio of the mean regression sum of squares divided by the mean error sum of squares;

m. $$R^2$$ – multiple R-squared; * – $$p<0.05$$; ** – $$p<0.01$$; *** – $$p<0.001$$

The linear regression model for the control group showed that body height is a statistically significant predictor variable on the SIRT RT test. An increase in body height of 1 cm contributes to a 2.12 ms increase in SIRT reaction time with a standard error of 0.62 ms. In the case of SIRT MT, it was observed that an increase in age by 1 year results in an increase in movement time by 5.11 ms, while SE is 2.05 ms. The only statistically significant predictor variable for CHORT RT is body height, which increases the reaction time with increasing height by 2.78 ms with a standard error of 1 ms. The number of correct answers is statistically significantly influenced by body height ($\beta$ = 0.54 ms, SE = 0.07 ms), body weight ($\beta$ = -0.65 ms, SE = 0.18 ms) and BMI index ($\beta$ = 2.40 ms, SE = 0.6 ms). In the HECOR test, it was observed that with a unit increase in age, the movement time in the group increased by 5.05, and the standard error is at the level of 2.21 ms. An increase of body height lengthens the reaction time by 2.79 ms with an SE of 1.27 ms. Body height is also a statistically significant predictor variable for SPANT MT, which results in an increase in movement time of 1.69 ms (SE = 0.77 ms) for a 1 cm increase in body height. The number of correct responses may be affected by height ($\beta$ = 0.62%, SE = 0.08%), weight ($\beta$ = -1.01%, SE = 0.20%) and BMI ($\beta$ = 2.97%, SE = 0.66%).

**Table 4:** Multiple Linear Regression for control group

Variable	Age		Body height		Body weight		BMI		RSE	m. *R*^2^	F	p
	*β*	SE	*β*	SE	*β*	SE	*β*	SE				
**SIRT**												
RT [ms]	-0.54	2.17	2.12**	0.62	-3.15	1.57	8.77	5.19	43.19	0.98	400.10	0.0001
MT [ms]	5.11*	2.05	-0.08	0.58	2.93	1.48	-6.16	4.90	40.76	0.96	153.00	0.0001
**CHORT**												
RT [ms]	3.03	3.54	2.78*	1.00	-1.86	2.56	10.42	8.46	70.42	0.99	632.30	0.0001
MT [ms]	3.72	2.26	0.64	0.64	2.04	1.63	-6.25	5.40	44.96	0.96	155.70	0.0001
c.r. [%]	-0.30	0.25	0.54***	0.07	-0.65***	0.18	2.40***	0.60	4.99	0.99	2376.00	0.0001
**HECOR**												
RT [ms]	0.35	2.69	1.56	0.76	-2.50	1.94	12.72	6.43	53.49	0.98	378.90	0.0001
MT [ms]	5.05*	2.21	-0.04	0.63	3.03	1.60	-4.59	5.29	44.03	0.97	208.60	0.0001
**SPANT**												
RT [ms]	1.39	4.47	2.79*	1.27	-4.92	3.23	17.39	10.68	88.95	0.98	277.50	0.0001
MT [ms]	1.51	2.73	1.69*	0.77	-0.75	1.97	-1.63	6.53	54.37	0.96	134.80	0.0001
c.r. [%]	-0.31	0.28	0.62***	0.08	-1.01***	0.20	2.97***	0.66	5.48	0.99	2073.00	0.0001

$\beta$ – unstandardized beta; SE – standard error; RSE – Residual Standard Error;

F – the ratio of the mean regression sum of squares divided by the mean error sum of squares;

m. $$R^2$$ – multiple R-squared; * – $$p<0.05$$; ** – $$p<0.01$$; *** – $$p<0.001$$

## Discussion

The main purposes of this study were to characterize the psychomotor abilities of male rugby players considering hypothetical correlated factors and to compare differences between professional rugby players and students of physical education.

The research shows that better SIRT RT was obtained in the control group, where the difference between the rugby players was calculated at the level of 29 ms and was statistically significant. In the CHORT RT, HECOR RT and SPANT RT tests, statistically significant differences were not observed. Comparing the results with those from previous studies using the computerised Test2Drive system, better reaction times in the SIRT test for the non-training group (n = 40) were also observed for tests of psychomotor abilities of Special Forces candidates (n = 48) [35], where the difference between groups was 17.6 ms and it was statistically significant. For the other tests (CHORT, HECOR and SPANT), as in the current study, the differences between groups were not statistically significant.

Contrary to our results, however, several studies showed that athletes had better reaction times than non-trainers [6, 10, 36–39]. In a previous scientific paper by Przednowek et al. [10], where 40 professional handball players from the Superliga, 1st and 2nd league of the Polish men’s handball were investigated, it was found that handball players achieved statistically significantly shorter reaction times in all tests performed (SIRT, CHORT, HECOR and SPANT) than the non-training group (n = 50). Mahesh et al. [36], using computerised tests (direct RT) in their study, compared reaction time in response to a simple visual signal of 50 badminton players with 50 no-training people. Based on the results, it was found that those involved in sports had better reaction time compared to the rest of the participants. Simple reaction time was also discussed by Ghuntla et al. [37], who, on the basis of tests compared the reaction time of 50 basketball players with a 50-person control group and found that those involved in sport reacted faster to a simple visual stimulus than the non-athletes. Simple reaction time was also a topic of interest by Kuan et al. [38], who examined 114 athletes in different sports (football, basketball, badminton, hockey, squash, volleyball) and 114 secondary students between 13-16 years old. According to their results, there was a statistically significant shorter reaction time in the group of athletes. Atan and Akyol [6] conducted a study in several sports groups, where the mean age was about 16 years old, and comparing their reaction time results with the control group of non-athletes and concluded that the simple reaction time of non-athletes has been statistically significantly higher than that of most athletes. Also, in the study by Seidel and Ragert [39], the simple reaction time in the group of non-athletes was statistically significantly longer than in football and handball players.

When comparing the movement time of rugby players with the control group (students of PE), the shorter movement time in the SIRT, CHORT, and HECOR tests was characterized by rugby players. The differences observed in the SPANT test weren’t statistically significant. Considering the results of psychomotor abilities using Test2Drive computer tests, Paśko et al. [35] noticed that in Special Forces candidates who have completed the first (fitness tests) and the second (mountain survival camp) of the selection stages, movement time was significantly shorter in all tests than in the control group represented by non-athletes. The highest differences were determined in the spatial anticipation test (SPANT, d = 166.2 ms), hand-eye coordination test (HECOR, d = 139.3 ms) and choice reaction time (CHORT, d = 134.0 ms). In the same study, a comparison was also conducted between a control group and a group of athletes (football, volleyball and handball players), where the athletes had a statistically significantly shorter movement time in performed tests. Statistically significant shorter movement time, compared to non-athletes, was also characterised by handball players [10], and the differences in the SIRT, CHORT, HECOR and SPANT tests were at the $\alpha <0.001$ level.

Other articles on the assessment of the psychomotor abilities of athletes, it was also noted that sports training can significantly improve reaction or movement time [20, 35, 39–42]. Seidel and Ragert [39] showed that even short-term, high-intensity exercise could improve movement time. Kuan et al. [38] confirmed that involvement in sports can improve eye-hand reaction time and anticipation time responses. The article by Zemkova and Hamar [40] showed that athletes who practice various team sports games (ice hockey, soccer, basketball, and volleyball) achieved much shorter reaction times in the agility test than physical education students. Ozmerdivenli et al. [41] have observed a significant difference in the reaction time test to visual and auditory stimuli between 100 physically active students from Physical Education and Sports College and 100 physically non-active students from the Faculty of Science and Literature of Fırat University. Akarsu et al. [42] showed that eye-hand reaction time was higher in the non-athlete group, and the difference between groups was statistically significant. Millard et al. [20] also observed that Premier League rugby players were statistically significantly better than non-athletes regarding visual coordination. Rugby players had 23% better hand-eye coordination abilities compared to the non-athlete group.

In the case of correct answers, in the CHORT and SPANT tests, no statistically significant differences were found between the rugby players and the control group. Likewise, in the article by Przednowek et al. [10], the differences in the percentage of correct answers between handball players and students were statistically insignificant. In turn, Paśko et al. [35], statistically significant differences were observed between the group of athletes (football, volleyball, and handball players), and the control group consisted of students from the University of Rzeszow only in the test evaluating reaction time with choice (CHORT).

Linear regression analysis for rugby players showed that the simple reaction time depends on body height. The slope of the regression line shows that the higher the body height value, the longer the SIRT response time. Body height is also a predictor of the reaction time test CHORT. The reaction time increases with increasing body height. In the HECOR test, body height correlates with reaction time and movement time. The increase of body height leads to a lengthens of reaction time and movement time in the HECOR test. Linear regression also showed a relationship between body height and the correct answers in the CHORT test. The analysis showed that as the body height increases, the number of correct answers also has been increasing.

Meanwhile, correct answers in the SPANT test depend on body height and body weight. The increase in body height causes an increase in the number of correct answers, while an increase in body weight decreases the level of choosing correct answers. In a linear regression analysis of the control group, it was also observed that reaction time in the SIRT test depends on body height. In addition, it has been shown that the older the participants, the longer the movement time. In the CHORT test, reaction time is determined by body height, while more correct responses are statistically significantly affected BMI, body weight and body height. In this group of subjects, the estimated percentage of correct answers increased by almost 3% with the individual increase in the BMI value. A much smaller but positive effect on c.r. [%] was also had by the body height of the subjects ($\beta =0.62$), and taller people made fewer mistakes. The higher body weight of persons in this group negatively affected the number of correct answers in the CHORT test ($\beta =-1.01$). In the case of the HECOR test, it was only observed that the older the participants, the longer the movement time. Higher body height was also shown to result in lower reaction time, movement time and an increased number of correct responses in the SPANT test. In addition, a higher number of correct responses is conditioned by a lower body mass. It is worth noting that a higher number of correct answers in the SPANT test is also dependent on a higher BMI. It was confirmed by Sudheer et al. [43] who showed that there is a correlation between various somatic features, such as body height and body weight and visual reaction time. The analysis also showed that as body height and body weight increase, the reaction time is longer. The same conclusions were made by Grewal et al. [44] who proved a longer reaction time in a group of overweight people than in people with normal body weight. Also the results of Ngo et al. [45] research showed that the ratio of body height and body mass (BMI) below the optimal range negatively affects on visual reaction time in group of people with underweight. Thus maintaining an optimal BMI, neither underweight nor overweight/obesity is highly recommended. Indeed, a meaningful relationship between BMI and reaction time (visual and auditory) in both men and women has been proved [29, 30, 44, 46–48]. Overweight or obese people had longer reaction times than normal body-weight people.

Given the above and the results of conducted analyses, it can be concluded that the players playing at the level of the Polish national team in Rugby were characterized by statistically significantly shorter movement times compared to the control group. Differences in movement time observed in the tests of simple reaction time (SIRT), reaction time with choice (CHORT) and eye-hand coordination (HECOR) may indicate the development and formation of these abilities during the sport ontogeny of rugby players or their training. Considering that the assessment of reaction time in children of different ages is useful for talent identification [40] and can be a good indicator of performance in reactive sports [37], one of the elements that should be assessed and monitored from the early stages of selection, and then over the next few years of career development, are the psychomotor abilities of the players. The analysis of visual reaction time and visual anticipation time can also be significant in a training programme and can lead to improved sports performance [38]. Because the accuracy of sensorimotor parameters measurements is influenced by many [40] factors, it may also be essential to assess anthropometric parameters, body composition or physical fitness of rugby players. The data collected in this way, additionally taking into account the position on the pitch and the effectiveness of the game, may in the future be helpful for coaches in choice of rugby players and assigning them appropriate tasks on the pitch.

Limitations of the study are relate to the small size of the research groups. The small size of the group of rugby players prevents to make a classification on position. In the future, the study could be extended to compare the psychomotor abilities of players by position on the pitch, as different tasks and player characteristics characterise them. In addition, it would be worthwhile to focus on analysing the relationship between psychomotor abilities and playing efficiency. The test2drive system, which we used, made it possible to assess the simple reaction time (RT), which is the time between the appearance of the stimulus and the removal of the finger from the START button, and the movement time (MT), which was measured from the moment of removing the finger by the subject to indicating the reaction area. Therefore, it isn’t always possible to refer the obtained results to other scientific publications [34]. The reaction time, consisting of the time measured from the appearance of the stimulus to the initiation of movement and the movement time [49], was not always distinguished in other works.

## Conclusions

The following conclusions were formulated based on the analysis of the data. First, the rugby players had shorter movement time in each psychomotor test than the physical education students, except for the SPANT test. Second, the control group achieved shorter reaction times in the SIRT test, while the other tests showed no statistically significant differences. This may indicate that reaction times in more complex tasks may be influenced by individual characteristics. Third, multiple Linear Regression analysis showed that the body height of rugby players was a significant predictor variable for simple reaction time, choice reaction time and hand-eye reaction time. Fourth, the height of the rugby players also significantly affected movement time in the eye-hand coordination test and the number of correct responses in the choice reaction time and spatial anticipation tests. Finally, the subjects’ body weight was a significant predictor variable for the correctness of the responses in the choice reaction time and spatial anticipation tests. For non-trainees, the number of correct indications was additionally statistically significantly influenced by BMI.

## Data Availability

The data used to support the findings of this study are available from the corresponding author upon request. The data sets generated and analysed during the current study are available.
